# Scrub Typhus among Febrile Children in a Tertiary Care Center of Central Nepal: A Descriptive Cross-sectional Study

**DOI:** 10.31729/jnma.6166

**Published:** 2021-05-31

**Authors:** Isha Bhandari, Kalpana Karmacharya Malla, Pukar Ghimire, Bibek Bhandari

**Affiliations:** 1 Department of Pediatrics, College of Medical Sciences and Teaching Hospital, Bharatpur, Chitwan, Nepal; 2 Department of Internal Medicine, College of Medical Sciences and Teaching Hospital, Bharatpur, Chitwan, Nepal

**Keywords:** *children*, *epidemiology*, *scrub typhus*

## Abstract

**Introduction:**

Scrub typhus is a mite borne infectious disease caused by Orientia tsutsugamushi, obligate intracellular bacteria, transmitted by chigger mites. Scrub typhus is an emerging febrile illness with clinical suspicion being the only key to diagnosis. This study was conducted to find out the prevalence of Scrub typhus among febrile children in a tertiary care center of central Nepal.

**Methods:**

A descriptive cross-sectional study was conducted from January 2018 to December 2019 in the pediatric inpatients of a tertiary care hospital after obtaining ethical clearance from Institutional Review Committee of Institute (Reference number 2020-105). Convenient sampling method was used. Data was analyzed using Statistical Packages for the Social Science version 16. Point estimate at 95% Confidence Interval was calculated along with frequency and proportion for binary data.

**Results:**

Out of 1024 febrile patients, prevalence of scrub typhus among febrile children was 55 (5.37%) (3.66-7.08 at 90% Confidence Interval). Of 55 patients, mean age was 9.2 years with 52 (94.6%) of cases diagnosed between July to November. Among 55 patients, other symptoms were vomiting 33 (60%), headache 22 (40%), abdominal pain 19 (34.5%), cough 15 (27.3%), nausea (25.5%), seizure 11 (20 %), and dyspnea 6 (10.9%). Major clinical signs was lymphadenopathy 29 (52.7%). Major complication noted was meningitis 11 (20%).

**Conclusions:**

The prevalence of scrub typhus is considerably high during July to November so it should be considered as a differential diagnosis of fever particularly in this period.

## INTRODUCTION

Scrub typhus is a mite borne infectious disease caused by Orientia tsutsugamushi, obligate intracellular bacteria, transmitted by chigger mites. Once considered localized to Asia-Pacific, it was later documented outside Asia-Pacific, first in Chile.^1^ It is now a major cause of fever in Asia with one million infections annually.^2^ In Nepal the major outbreak were reported after the devastating earthquake on 2015.^3^

Diagnosis requires a high index of suspicion as signs and symptoms are non-specific like fever, rash, cough, headache, myalgia, lymphadenopathy, vomiting and abdominal pain.^4^ Failing to treat on time leads to complications like multiorgan damage, pneumonitis, acute respiratory distress syndrome, myocarditis, septic shock, meningoencephalitis, including mortality.^5^

The aim of this study is to find the prevalence of scrub typhus among febrile child and to characterize the signs, symptoms, and complications which will help in early diagnosis and management with appropriate drugs to prevent morbidity and mortality.

## METHODS

This is a descriptive cross-sectional study conducted at College of Medical Sciences Teaching Hospital (COMSTH) over a period of 2 years (January 2018 to December 2019) after ethical clearance from Institutional Review Committee team of College of Medical Sciences Teaching Hospital (Reference no. 2020-105). All the children with febrile illness having complete data during period from January 2018 to December 2019 were included in the study. The febrile children having incomplete or missing data were excluded from the study. Convenient sampling method was used. The sample size was calculated using the formula,


$$\begin{array}{ll} \text{n} & \text{= }{\text{Z}}^{\text{2}}\times \text{p}\times \text{q}/{\text{e}}^{\text{2}}\\ & \text{= }{\left(1.645\right)}^{\text{2}}\times 0.032\times 0.968/{(\text{0.01)}}^{\text{2}}\\ & \text{= }838 \end{array}$$

Where,

n = minimum required sample size
p = prevalence from previous study, 3.2%^6^
Z = 1.645 at 90% Confidence Interval (CI)
q = 1-p
e = margin of error, 1%

The minimum required sample size was 838. Adding 10% non-response rate, a sample of 922 was reached. But we included 1024 febrile children aged 1-15 years in the study. Fifty five cases diagnosed as Scrub Typhus during the study period. Clinical symptoms and signs were noted along with lab parameters and complications. AKI was defined by an increase in serum creatinine by >0.3mg/dl within 48 hours or urine volume <0.5ml/kg/hr for 6 hours as per AKI Network definition.^7^ Hyponatremia was defined as serum sodium <135mEq/L. Acute hepatitis was defined as elevation of serum transaminase more than 2 times the normal upper limit. Meningitis was diagnosed on the basis of presence of signs of meningeal irritation and CSF analysis demonstrating an increase in cell count, elevated protein and decreased sugar. Leukocytosis and leucopenia were defined as total leukocyte count more than 11,000/cumm and less than 4,000/cumm respectively. All data obtained were entered to an Excel worksheet and were analyzed using Statistical Package for Social Sciences version 16.0.

## RESULTS

Among 1024 febrile patients, 55 (5.37%) (3.66-7.08 at 90% CI) was confirmed to be Scrub typhus. Out of 55 patients diagnosed as cases of scrub typhus, the mean age was 9.2+3.9 years. Of the 55 patients, 30 (54.5%) were females and 25 (45.5%) were male (Table 1).

**Table 1:** Male female distribution of patients with Scrub Typhus (n = 55).

Variable	n (%)
Sex	
Male	25 (45.5)
Female	30 (54.5)
Total	55 (100)

About 53 (96.4%) of the patients were Hindus. Scrub typhus is seasonal and 94.6% of cases were diagnosed between the months of July to November. Fever was present in all 55 (100%) among the patients. Other symptoms in decreasing order of frequency were vomiting 33 (60%), headache 22 (40%), abdominal pain 19 (34.5%), cough 15 (27.3%), nausea 14 (25.5%), seizure 11 (20.0%) and shortness of breath 6 (10.9%). On examination, edema was seen in 13 (23.6%) of patients followed by hepatomegaly in 10 (18.2%) , splenomegaly in 6 (10.9%) of the patients. Eschar was demonstrated in 6 (10.9%) of patients, 6 (10.9%) of patients were pale and 2 (3.6%) were icteric (Table 2).

**Table 2:** Demographic and clinical characteristics among patients of Scrub typhus (n = 55).

Religion	n (%)
Hindu	53 (96.4)
Buddhist	2 (3.6)
**Month of year at presentation**	
July	6 (10.9)
August	14 (25.5)
September	15 (27.3)
October	12 (21.8)
November	5 (9.1)
Others	3 (5.4)
**Symptoms**	
Fever	55 (100)
Vomiting	33 (60)
Headache	22 (40)
Pain abdomen	19 (34.5)
Cough	15 (27.3)
Nausea	14 (25.5)
Seizure	11 (20)
SOB	6 (10.9)
**Clinical Findings**	
Lymphadenopathy	29 (52.7)
Edema	13 (23.6)
Hepatomegaly	10 (18.2)
Splenomegaly	6 (10.9)
Eschar	6 (10.9)
Pallor	6 (10.9)
Icterus	2 (3.6)

Hemoglobin level was less than 10gm/dl in 18 (32.7%) of the patients. Nine (16.4%) of patients had leukocytosis and 8 (14.5%) had leucopenia. Thrombocytopenia was common and was found in 35 (63.63%) with 2 (3.6%) of the patients had the platelet count below 50,000. Hyponatremia was noted in 28 (50.9%) and Hypokalemia in 7 (12.7%). Transaminitis with raised AST was seen in 25 (45.5%) of cases and raised ALT was seen in 19 (34.5%) of cases. CRP was positive in 44 (80.0%) of cases (Table 3).

**Table 3:** Lab abnormalities among the Scrub typhus patients (n = 55).

Lab parameters	n (%)
Hb <10gm/dl	18 (32.7)
Total Leucocyte count	
Less than 4000	8 (14.5)
4000 to 11000	38 (69.1)
More than 11000	9 (16.4)
Thrombocytopenia	35 (63.6)
1 to 1.5 lakhs	21 (38.2)
50000 to 1 lakh	12 (21.8)
Less than 50000	2 (3.6)
Hyponatremia(Na<135mEq/L)	28 (50.9)
Hypokalemia (K <3.5)	7 (12.7)
AST 2X UNL or more	25 (45.5)
ALT 2X UNL or more	19 (34.5)
Positive CRP	44 (80)

**Figure 1.**
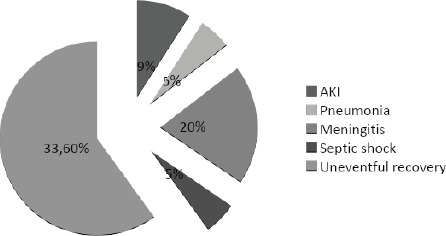
Complication among the patients of Scrub typhus.

Of 55 patients, 5 (9.1%) developed AKI but none required hemodialysis. Eleven (20.0%) had meningitis, 3 (5.5%) developed pneumonia and 3 (5.5%) of them were complicated with sepsis. Thirty three (60.0%) of the patients had uneventful recovery with no complications. There were no mortalities (Figure 1).

Forty nine (89.1%) of the patients were cured with a single agent, with Doxycycline being the most commonly used drug in 28 (50.9%) patients, followed by Chloramphenicol in 12 (21.8%) and Azithromycin in 9 (16.4%) of the patients. Six (11.0%) patients required combination therapy among which 5 (9.1%) patients received a combination of Doxycycline and Azithromycin while 1 (1.8) patient was treated with a combination of Azithromycin and Chloramphenicol (Table 4).

**Table 4:** Distribution of antibiotic received.

Antibiotics received	n (%)
Single agent	
Doxycycline	28 (50.9)
Chloramphenicol	12 (21.8)
Azithromycin	9 (16.4)
Combination agents	
Doxycycline and Azithromycin	5 (9.1)
Azithromycin and Chloramphenicol	1 (1.8)

## DISCUSSION

Scrub typhus was the commonly diagnosed disease in the Asia-Pacific region but recently it has encroached most of the world and now is also seen frequently in Nepal. However, data on Scrub Typhus in pediatric population in our country is still scarce.

The mean age at presentation was 9.2 years in our study which was similar to that reported by other author.^8,9^ In contrast to other studies that showed the disease to be predominant in males, our study population showed female predominance. In our part of the world, household and farming activity are done mostly by female, even as children, exposing themselves more to the bite of the mite.^8,10^ Study from south India has also reported a higher incidence in females.^8^

Similar to what is reported in a study by Pathak S, et al. in Eastern Nepal, most of the cases occurred in the month of August, September, and October when the climate is hot and humid.^8^ The months of peak incidence is found to be different in different parts of the world. In a study done in Chile, the disease peaked during the summer months of February and March.^11^

With exceptions of few studies, like in the study by Saleem, et al. where headache and myalgia were the most common manifestation followed by fever, most of other studies, including ours have shown fever to be almost universal in Scrub Typhus.^12,13^

Lymphadenopathy has been reported in a variable frequency in different studies. Our study found lymphadenopathy in 52.7% of patients which was similar to that reported by Mahajan, et al.^14^ But, few other studies have shown lymphadenopathy only in few number of patients. A study done in Odisha, Eastern India reported lymphadenopathy only in 2.97% whereas the incidence was 24% in a study done at TUTH.^15,16^

Other symptoms and signs such as headache ,seizure was noted in 40%, 20% respectively which was similar to the finding in study done at TUTH.

Typical eschar was documented only in 10.9% of the study population. Although eschar is considered as the typical finding in scrub typhus various studies have shown the finding in quite variable range 7% to 68%.^17^

Thrombocytopenia has always been reported as a common finding in Scrub Typhus by various studies.^9,18^ We also found platelets to be less than 1.5 lakhs in 63.6% of our patients. Lung has been shown to be a major organ for Scrub infection in humans and in animal infection models.^19^ The lung has also been shown to be a site of platelet biogenesis and a reservoir for hematopoietic progenitors, which is one of the pathogenesis of thrombocytopenia in Scrub Typhus.^20^

Hyponatremia is a common encounter in Scrub typhus. We found the occurrence to be 50.9%. The occurrence is however variable with 38% in TUTH and a range of 15-32% in studies from other countries of the world.^9,21^ Scrub Typhus has been explained as a disease that dysregulates immune and vascular response.^19^ Hyponatremia in scrub reflects increased vascularity and is a common finding in Rickettsial diseases.^22^ Cerebral salt wasting has also been reported as cause of hyponatremia in Scrub Typhus in few occasions.^23^

In our study population we found thrombocytopenia with raised CRP. This combination of lab abnormality has been observed in various other studies.^10,24^ This finding has always been a characteristic of scrub typhus in contrast to dengue, where thrombocytopenia is not always associated with raised CRP.

AKI as a complication of scrub typhus was found in 9.1% in our study and similar finding was noted in the study in children of Southern India by Kumar, et al.9 Renal complications in the form of AKI was the matter of study in different articles which showed AKI as the complication in as minimum as 2% to as high as 20%.^21,25^ AKI in scrub typhus is believed to be multifactorial in origin. Common causes accounted for AKI, in adult patients, are impaired renal perfusion because of shock or increased vascular permeability, rhabdomyolysis, vasculitis, thrombotic microangiopathy and acute tubular necrosis due to direct microbial invasion of renal tubules.^26,27^

CNS complication as meningitis was found in 20% which was comparable to another study done in our country where meningitis and meningoencephalitis were seen in 30.4%.^16^ The rate of complications in the form of sepsis, meningitis and pneumonia was found to be 5.5%, 20%, 5.5% respectively which was similar to findings by Chapagain RH, et al.^18^ All these complications in scrub typhus is due to the endothelial tropism of Orientia that can lead to vasculitis, that affects multiple organ, especially in severe cases.^28^ Pathological characteristics of fatal scrub typhus include diffuse interstitial pneumonia, hepatic lesions, meningoencephalitis, and coagulation disorders.^4^

The drugs used for the treatment were doxycycline, azithromycin and chloramphenicol. Of these, doxycycline was the most preferred one. We had observed good response with doxycycline in the study population. Other drugs were used when Doxycycline was not tolerated or when broad spectrum coverage was needed. Doxycycline alone was highly effective drug in our study which was used in 50.9% of the patients. Similar was the experience by Kumar, et al.^12^ Injectable Chloramphenicol was used in 21.8% of the cases depending on the severity of the disease process and the drug showed rapid response which was similar to the study done in Srilanka.^29^ Similar to our study the combination therapy with doxycycline and azithromycin was used by Chapagain RH, et al. in their study considering the management of coinfection and complication.^18^ Single centered study and small study population are the limitations of the present study. Large scale and multi-centered study are needed to localize the most common clinical signs and symptoms in scrub typhus in children.

## CONCLUSIONS

The prevalence of scrub typhus is considerably high during july to november so it should be considered as a differential diagnosis of fever particularly in this period. Scrub typhus has now emerged as one of the important differential of acute febrile illness in the pediatric population especially during certain period of the year. Nonspecific multisystem symptoms in patients with thrombocytopenia and increased CRP, hyponatremia, transaminitis with involvement of lungs, kidneys and brain in a febrile child should strike the diagnosis. Once diagnosed, this disease can be successfully treated with easily available antibiotics like doxycycline, azithromycin, chloramphenicol with rapid recovery of the patient.
